# Cardiovascular Effects of 4% Articaine with 1:100,000 versus 1:200,000 Epinephrine during Inferior Alveolar Nerve Block: A Prospective Randomized Double-Blind Clinical Study

**DOI:** 10.4317/jced.63739

**Published:** 2026-03-30

**Authors:** Marcele Cruz da Silva, Bruno Teixeira Gonçalves Rodrigues, Estephani Martins Barcellos de Carvalho, Thaís Pimentel de Sá Bahia, Fábio Gamboa Ritto, Paulo José d’Albuquerque Medeiros

**Affiliations:** 1 DDS, OMFS, MsC. Oral &amp; Maxillofacial Surgery, Pedro Ernesto University Hospital, Rio de Janeiro State University, Rio de Janeiro, Brazil; 2 DDS, OMFS. Oral &amp; Maxillofacial Surgery, Pedro Ernesto University Hospital, Rio de Janeiro State University, Rio de Janeiro, Brazil; 3 DDS, OMFS, PhD. Oral &amp; Maxillofacial Surgery, Pedro Ernesto University Hospital, Rio de Janeiro State University, Rio de Janeiro, Brazil; 4 DDS, M.D, PhD. Department of Oral and Maxillofacial Surgery, College of Dentistry, The University of Oklahoma, United States of America

## Abstract

**Background:**

Local anesthetic solutions containing epinephrine are widely used in oral surgery to prolong anesthesia and improve hemostasis; however, their sympathomimetic effects may influence cardiovascular parameters. Evidence comparing different epinephrine concentrations in articaine during third-molar surgery remains limited. This randomized clinical trial compared the cardiovascular effects and clinical performance of 4% articaine with epinephrine 1:100,000 (A100) and 1:200,000 (A200) during mandibular third-molar extraction.

**Material and Methods:**

A prospective, randomized, split-mouth, double-blind clinical trial was conducted in 40 healthy patients undergoing bilateral impacted mandibular third-molar extraction. Each participant received A100 on one side and A200 on the contralateral side in randomized order with a 15-day interval. Heart rate (HR), blood pressure (BP), and oxygen saturation (SpO2) were recorded at baseline, immediately after injection, and at 5 and 15 minutes. Intraoperative bleeding, pain (VAS), and need for supplemental anesthesia were also assessed. Paired statistical tests were applied ( = 0.05).

**Results:**

Eighty procedures were analyzed. Intraoperative bleeding incidence was similar between A100 (10%) and A200 (12.5%) (p = 1.000). Supplemental anesthesia was required in 3.8% of procedures, with no difference between concentrations (p = 1.000). HR increased with both formulations but was significantly higher with A100 at 5 and 15 minutes (p = 0.035 and p = 0.003). BP showed no significant differences at any time point. SpO2 remained within normal limits, with slightly higher values for A200 at intermediate times. Pain scores did not differ significantly.

**Conclusions:**

Both epinephrine concentrations in 4% articaine provided comparable anesthetic efficacy and hemostasis with minimal cardiovascular effects in healthy patients undergoing mandibular third-molar extraction. The 1:200,000 concentration produced a smaller heart-rate increase, suggesting a potential advantage in patients with cardiovascular risk, while 1:100,000 remains suitable when greater vasoconstriction is desired.

## Introduction

Effective local anesthesia is essential for the success and safety of oral surgical procedures. Local anesthetic solutions containing vasoconstrictors are routinely used in dentistry because they prolong anesthetic duration, reduce systemic toxicity, and improve intraoperative hemostasis (1). However, vasoconstrictors such as epinephrine exert sympathomimetic effects that may increase heart rate and blood pressure, making their concentration clinically relevant, particularly in patients with cardiovascular risk (2 , 3). In addition to pharmacological effects, procedural anxiety and surgical stress can trigger endogenous catecholamine release, leading to transient hemodynamic alterations during dental treatment (2 - 4). Inadequate anesthesia and intraoperative pain may further enhance this adrenergic response, potentially increasing cardiovascular load. For this reason, optimizing anesthetic efficacy while minimizing vasoconstrictor exposure remains an important consideration in oral surgery (1 - 4). Articaine is widely used in dental anesthesia due to its thiophene ring structure, which increases lipid solubility and facilitates diffusion through neural tissues (4 , 5). Commercial formulations commonly include epinephrine at concentrations of 1:100,000 or 1:200,000. The lower concentration is often recommended for patients with cardiovascular compromise, whereas 1:100,000 has demonstrated high anesthetic efficacy and hemostatic control in oral surgery with low incidence of adverse events (1). Recent clinical studies and systematic reviews have confirmed the cardiovascular safety of epinephrine-containing local anesthetics within recommended dose limits, even in medically compromised patients (6 , 7). Nevertheless, evidence comparing different epinephrine concentrations in articaine during oral surgical procedures remains limited and somewhat heterogeneous, particularly regarding cardiovascular responses and intraoperative bleeding control in healthy individuals undergoing third molar surgery (7 - 11). Therefore, the aim of this prospective, randomized, split-mouth, double-blind clinical trial was to compare the cardiovascular effects and clinical performance of 4% articaine with epinephrine 1:100,000 (A100) and 1:200,000 (A200) during inferior alveolar nerve block (IANB) for mandibular third molar extraction in health.

## Material and Methods

This study was designed as a prospective, randomized, split-mouth, double-blind clinical trial with a 1:1 allocation ratio with a convenience sample. Each participant underwent two surgical procedures for bilateral impacted mandibular third molar extraction, receiving 4% articaine with epinephrine 1:100,000 on one side and 1:200,000 on the contralateral side, in randomized order, with a 15-day interval between surgeries. The study protocol was approved by the Ethics Committee of the Pedro Ernesto University Hospital, Rio de Janeiro State University, Brazil (CAAE: 23255413.3.0000.5259; approval no. 496.117). All participants provided written informed consent prior to enrollment, and the study was conducted in accordance with the Declaration of Helsinki. Participant recruitment and data collection were performed in 2013, before the widespread requirement for prospective registration of dental randomized clinical trials in public registries; therefore, this study was not registered. Forty healthy patients (10 men and 30 women; mean age 20.6 years, range 14-32 years) requiring bilateral surgical extraction of impacted mandibular third molars were recruited from the Oral and Maxillofacial Surgery Service of Pedro Ernesto University Hospital between June and November 2013. Eligibility criteria included the presence of bilateral impacted mandibular third molars classified as Pell and Gregory classes 1A, 1B, 2A, or 2B, indication for surgical extraction, and absence of systemic disease. Exclusion criteria comprised systemic conditions contraindicating vasoconstrictor use (such as hypertension, uncontrolled hyperthyroidism, or cardiovascular disease), teeth classified as Pell and Gregory class III and/or C, and radiographic signs of high surgical complexity. All participants underwent clinical and panoramic radiographic evaluation before surgery. The sample size of 40 participants corresponded to the number of eligible patients treated during the recruitment period. The vasoconstrictor concentration administered at each surgery (epinephrine 1:100,000 or 1:200,000) was determined by coin toss immediately before anesthetic administration, and the allocation result was recorded for each session. In the first appointment, the right side was operated, and in the second appointment the contralateral side received the alternate anesthetic solution. Participants were blinded to the anesthetic formulation used in each procedure. A single surgeon performed all anesthetic injections and surgeries, while a second independent examiner, blinded to allocation, recorded cardiovascular parameters, intraoperative bleeding, and need for supplemental anesthesia. Statistical analyses were performed using coded data. Inferior alveolar, lingual, and buccal nerve blocks were performed using 2.7 mL of 4% articaine with epinephrine 1:100,000 or 1:200,000 (DFL®, Rio de Janeiro, Brazil), consisting of 1.8 mL for inferior alveolar nerve block and 0.9 mL for buccal infiltration. A standardized surgical technique was applied: a mucoperiosteal flap was elevated in an anteroposterior direction without extension beyond the external oblique ridge; osteotomy around the crown was performed followed by odontosection using surgical burs under copious sterile irrigation; tooth fragments were removed with a straight elevator; and the flap was repositioned and sutured with 3-0 silk. The primary outcome was cardiovascular response to anesthetic administration, assessed by heart rate (HR) and blood pressure (BP). Secondary outcomes included oxygen saturation (SpO2), intraoperative bleeding, and anesthetic efficacy (pain and need for supplemental anesthesia). HR, BP, and SpO2 were recorded using a multiparameter monitor (Dixtal®) at four time points: t0 (5 minutes before injection), t1 (immediately after injection), t2 (5 minutes after injection), and t3 (15 minutes after injection). Intraoperative bleeding was assessed by the blinded examiner using a dichotomous question ("Did bleeding interfere with the procedure?" yes/no). Pain was evaluated using a 10-cm visual analog scale (VAS) completed by the patient. Anesthetic efficacy was also assessed by the need for supplemental infiltration with 3% mepivacaine without vasoconstrictor. The null hypothesis was that articaine with epinephrine 1:100,000 and 1:200,000 would produce similar cardiovascular effects, hemostasis, and anesthetic efficacy. Paired statistical analyses appropriate for a split-mouth design were applied. McNemar's test was used to compare intraoperative bleeding and supplemental anesthesia. Paired t-tests were used for heart rate and blood pressure comparisons, and the Wilcoxon signed-rank test was used for SpO2 and VAS scores. The significance level was set at 5% ( = 0.05).

## Results

A total of 80 surgical procedures were performed in 40 participants in a split-mouth design, with each patient receiving both anesthetic formulations. Intraoperative bleeding interfering with the surgical procedure occurred in 9 of 80 extractions (11.3%). The incidence was similar between concentrations: 4 of 40 procedures (10%) with epinephrine 1:100,000 and 5 of 40 (12.5%) with epinephrine 1:200,000 (p = 1.000). Only one participant exhibited excessive bleeding under both conditions. Overall, bleeding was considered within normal limits in 32 of 40 participants (80%). Supplemental anesthesia was required in 3 procedures (3.8%), corresponding to one case with epinephrine 1:100,000 and two with epinephrine 1:200,000 (p = 1.000). No participant required reinjection on both sides. Intraoperative pain scores assessed by the visual analog scale (VAS) showed no statistically significant difference between concentrations (p = 0.974). Scores ranged from 0 to 6 with epinephrine 1:100,000 and from 0 to 3 with epinephrine 1:200,000 (Table 1).

Table 1

Regarding hemodynamic parameters, systolic blood pressure (SBP), diastolic blood pressure (DBP), and pulse pressure (PP) showed no statistically significant differences between anesthetic concentrations at any measurement time point (t0-t3). The greatest divergence was observed for PP at t2 (5 minutes after injection), when mean PP increased by 3.8 mmHg from baseline with epinephrine 1:100,000, whereas no change was observed with epinephrine 1:200,000 (p = 0.075) (Tables 2,3,4).

Table 2

Table 3

Table 4

**Figure 1:**
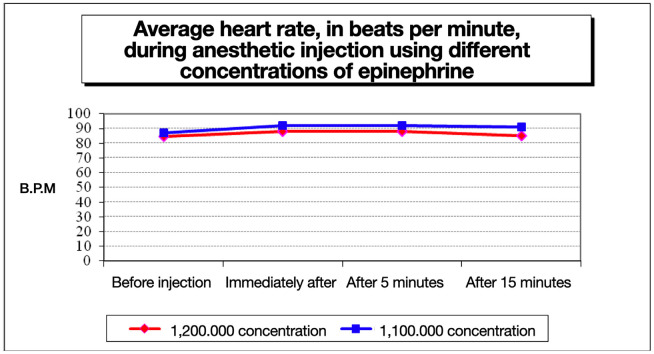
Mean heart rate recorded before anesthetic injection, immediately after injection, and at 5 and 15 minutes following administration of 4% articaine with epinephrine at concentrations of 1:100,000 and 1:200,000 during inferior alveolar nerve block.

Heart rate increased after anesthetic administration with both concentrations. Although the magnitude of change was clinically small, statistically significant differences between concentrations were detected at t2 (p = 0.035) and t3 (p = 0.003) (Fig. 1).

1

**Figure 2:**
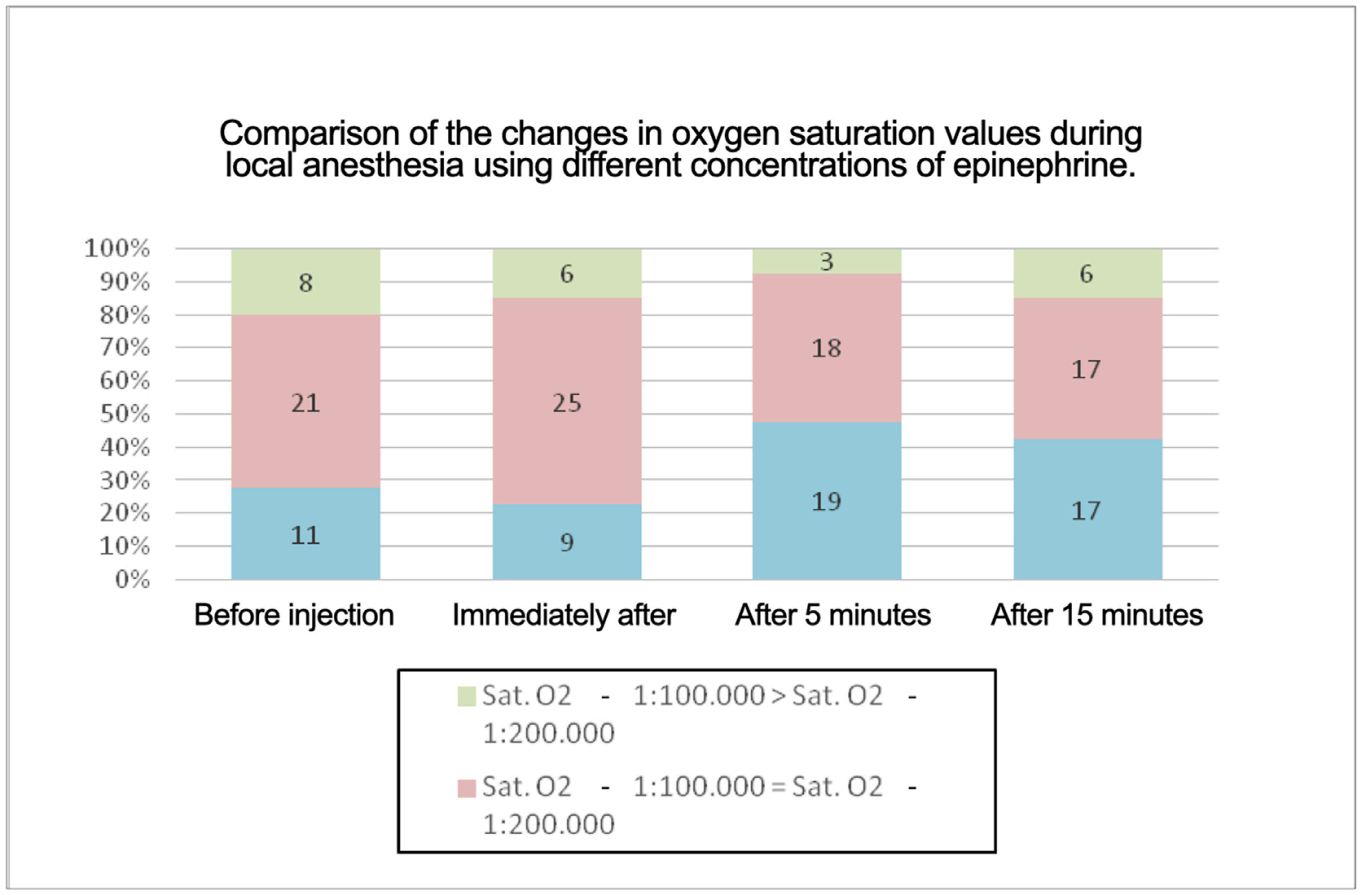
Mean SpO2, measured before anesthetic injection, immediately after injection, and at 5 and 15 minutes following administration of 4% articaine with epinephrine at concentrations of 1:100,000 and 1:200,000 during inferior alveolar nerve block.

The 1:100,000 formulation produced a greater elevation in heart rate and a slower return toward baseline values compared with the 1:200,000 formulation. Oxygen saturation remained within normal physiological limits in both groups. Statistically significant differences were observed at t2 and t3, with slightly higher values recorded with epinephrine 1:200,000. Measurements ranged from 97% to 100% with epinephrine 1:100,000 and from 99% to 100% with epinephrine 1:200,000 (Fig. 2).

2

## Discussion

The incorporation of vasoconstrictors into dental local anesthetic solutions remains a cornerstone of contemporary oral surgical practice because it prolongs anesthetic duration, improves hemostasis, and reduces systemic absorption (10 - 15). Nevertheless, epinephrine exerts sympathomimetic effects that may influence cardiovascular parameters, making concentration selection clinically relevant, particularly in patients with cardiovascular risk (3). Recent systematic reviews and clinical studies have confirmed the overall safety of epinephrine-containing dental anesthetics within recommended doses, while emphasizing the importance of evaluating concentration-dependent hemodynamic responses (7 , 8). Third-molar extraction continues to represent a reliable clinical model for investigating anesthetic efficacy and systemic responses because of its bilateral symmetry, procedural standardization, and reproducibility (9 , 11). In this context, articaine is particularly suitable due to its high lipid solubility, rapid onset, and favorable safety profile (4). Importantly, it is commercially available with epinephrine at both 1:100,000 and 1:200,000 concentrations, allowing clinically relevant comparisons under controlled conditions. The split-mouth randomized design adopted in the present study further minimized interindividual variability and strengthened internal validity. The present findings demonstrated statistically significant increases in heart rate at 5 and 15 minutes after injection with the 1:100,000 formulation compared with 1:200,000 epinephrine. Although statistically detectable, these changes were small in magnitude and remained within physiological limits, indicating minimal clinical impact. Blood pressure values showed only minor fluctuations without significant differences between concentrations, reinforcing the overall cardiovascular stability of both anesthetic solutions. These observations are consistent with contemporary evidence indicating that recommended epinephrine doses in dental anesthesia produce limited hemodynamic disturbance in healthy individuals (1 , 6 - 8 , 10 - 12). Oxygen saturation values were slightly higher with the A200 formulation at intermediate time points; however, all measurements remained within normal physiological ranges, and the differences are unlikely to be clinically meaningful. Anesthetic efficacy was high in both groups, with very low need for supplemental anesthesia. Likewise, intraoperative bleeding was comparable between concentrations and did not interfere with surgical performance. These findings support previous randomized trials reporting similar clinical effectiveness between A100 and A200 epinephrine in articaine for third-molar surgery (9). Historically, higher epinephrine concentrations such as 1:50,000 and 1:80,000 were investigated to maximize vasoconstriction; however, concerns regarding cardiovascular stimulation limited their clinical use (2 , 3 , 12). Current dental practice has therefore converged on A100 and A200 as the most appropriate balance between efficacy and safety (1 - 5). The present study adds evidence supporting this paradigm by demonstrating comparable anesthetic performance and minimal cardiovascular differences between these concentrations in a controlled surgical model. From a clinical perspective, both formulations achieved the fundamental objectives of local anesthesia in oral surgery: effective analgesia, adequate hemostasis, and cardiovascular stability. The slightly lower heart-rate response observed with 1:200,000 epinephrine may be advantageous when treating patients with cardiovascular vulnerability, whereas 1:100,000 may remain preferable when maximal hemostasis is desired. Thus, concentration selection can be individualized without compromising anesthetic success. Some limitations should be acknowledged. Although data collection was completed in 2013, subsequent stages of data processing and reporting were delayed by institutional constraints affecting the public university setting in which the study was conducted, including periods of financial crisis, funding shortages, and institutional strikes, as well as the later impact of the COVID-19 pandemic. The dataset was subsequently recovered, verified, and reanalyzed to ensure methodological rigor and compliance with current CONSORT reporting standards prior to submission.

## Conclusions

The present prospective, randomized, double-blind clinical study demonstrated that 4% articaine combined with either 1:100,000 or 1:200,000 provide comparable anesthetic efficacy and hemostasis with minimal cardiovascular effects in healthy patients undergoing mandibular third-molar extraction. Both concentrations can therefore be considered safe and effective for routine oral surgical procedures.

## Figures and Tables

**Table 1:** Experimental crossover study demonstrating the reported pain score (VAS).

Pain score -	Total (%)		Pain score - 1:200.000			
1:100.000			0	1	2	3
0	22	(55.0)	13	4	2	3
1	6	(15.0)	3	2	1	0
2	8	(20.0)	1	2	3	2
3	2	(5.0)	2	0	0	0
5	1	(2.5)	1	0	0	0
6	1	(2.5)	1	0	0	0
Total (%)	40	(100.0)	21 (52.5)	8 (20.0)	6 (15.0)	5 (12.5)

Note: P-value Wilcoxon test = 0,974

**Table 2:** Descriptive statistics and paired t-test evaluating pulse pressure.

	Pulse Pressure (mmHg)			
Descriptive	in relation to the moment of solution injection			
Statistics	Before	Immediately after	After 5-minutes	After 15-minutes
	(t0)	(t1)	(t2)	(t3)
1:100.000 Concentration				
Mean	53.8	55.9	57.6	57.7
Standard-deviation	12.4	11.0	10.0	11.4
Minimum	25.0	33.0	40.0	38.0
Median	54.0	56.5	57.0	57.5
Maximum	89.0	77.0	79.0	83.0
p-value from the paired t-test (comparison with the previous time point)				
1:200.000 Concentration				
Mean	54.8	55.5	54.8	55.7
Standard-deviation	10.3	12.5	11.0	12.7
Minimum	40.0	32.0	35.0	36.0
Median	52.0	53.5	52.0	54.5
Maximum	75.0	81.0	79.0	89.0
p-value from the paired t-test (comparison with the previous time point)				
p-value from the paired t-test (comparison between the concentrations)	0.599	0.812	0.075	0.229

2

**Table 3:** Descriptive statistics and paired t-test evaluating systolic blood pressure.

	Systolic Blood pressure (mmHg)			
Descriptive	in relation to the moment of solution injection			
Statistics	Before	Immediately after	After 5-minutes	After 15-minutes
	(t0)	(t1)	(t2)	(t3)
1:100.000 Concentration				
Mean	123.9	126.4	122.5	125.3
Standard-deviation	15.1	18.1	11.2	12.9
Minimum	100.0	90.0	100.0	96.0
Median	122.0	126.5	122.0	122.0
Maximum	163.0	197.0	144.0	153.0
p-value from the paired t-test (comparison with the previous time point)				
1:200.000 Concentration				
Mean	124.2	125.5	121.4	123.3
Standard-deviation	14.4	12.2	12.7	15.1
Minimum	98.0	103.0	102.0	97.0
Median	123.0	125.5	121.5	124.5
Maximum	152.0	148.0	148.0	163.0
p-value from the paired t-test (comparison with the previous time point)				
p-value from the paired t-test (comparison between the concentrations)	0.862	0.704	0.526	0.341

3

**Table 4:** Descriptive statistics and paired t-test evaluating diastolic blood pressure.

	Diastolic Blood pressure (mmHg)			
Descriptive	in relation to the moment of solution injection			
Statistics	Before	Immediately after	After 5-minutes	After 15-minutes
	(t0)	(t1)	(t2)	(t3)
1:100.000 Concentration				
Mean	70.1	70.5	65.0	67.5
Standard-deviation	8.6	17.1	5.0	6.5
Minimum	53.0	47.0	54.0	46.0
Median	71.0	68.5	64.0	68.0
Maximum	86.0	164.0	77.0	80.0
p-value from the paired t-test (comparison with the previous time point)				
1:200.000 Concentration				
Mean	69.4	70.0	66.7	67.7
Standard-deviation	8.9	9.9	7.3	9.1
Minimum	51.0	54.0	52.0	49.0
Median	69.0	69.5	66.5	68.0
Maximum	86.0	109.0	83.0	95.0
p-value from the paired t-test (comparison with the previous time point)				
p-value from the paired t-test (comparison between the concentrations)	0.656	0.831	0.148	0.918

4

## Data Availability

The datasets used and/or analyzed during the current study are available from the corresponding author.
